# THE PROTECTIVE ROLE OF FLAVONOIDS IN AMNESIA -SYSTEMATIC REVIEW

**DOI:** 10.12688/f1000research.164798.1

**Published:** 2025-07-21

**Authors:** Regan Mujinya, Swase Dominic Terkimbi, Victor Otu Oka, Monday Etukudo Ekon, Makena Wusa, Elna Owembabazi, Patrick Aja Maduabuchi, Daniel Udofia Owu

**Affiliations:** 1 Department of Physiology, Faculty of Biomedical sciences, Kampala International University - Western Campus, Bushenyi, Western Region, P.O Box. 71, Uganda; 2 Department of Physiological, Equator University of Science and Technology, Masaka, Masaka, B.O. `box 21, Uganda; 3 Department of Biochemistry, Faculty of Biomedical Sciences, Kampala International University - Western Campus, Bushenyi, Western Region, B.O Box. 71, Uganda; 4 Department of Human Anatomy, Faculty of Biomedical sciences, Kampala International University - Western Campus, Bushenyi, Western Region, P.O.Box 71, Uganda

**Keywords:** Flavonoids, Amnesia, Cognitive Enhancement, Oxidative Stress, Neuroprotection

## Abstract

**Background:**

Amnesia, a debilitating memory disorder, often arises from neurodegenerative diseases, oxidative stress, and synaptic dysfunction. Current therapies offer limited efficacy and are associated with adverse effects, prompting exploration of safer neuroprotective agents. Flavonoids, abundant in fruits, vegetables, and medicinal plants, have emerged as promising candidates due to their antioxidant, anti-inflammatory, and neurotrophic properties.

**Objective:**

This systematic review critically evaluated experimental evidence on the protective role of flavonoids in amnesia, highlighting their mechanisms of action, therapeutic outcomes, and translational potential.

**Methods:**

A comprehensive literature search was conducted using structured search strategies across the Web of Science, Scopus, and PubMed databases. Studies published between 2010 and 2024 were screened according to PRISMA 2020 guidelines. Only original experimental research investigating flavonoid interventions in animal models of amnesia was included.

**Results:**

Out of 593 retrieved articles, 25 studies met the inclusion criteria. Behavioral assessments predominantly employed the Morris Water Maze, Novel Object Recognition Test, and Y-Maze to evaluate cognitive performance. Flavonoid interventions consistently improved memory functions, reduced oxidative stress markers (MDA), enhanced antioxidant defenses (GSH, SOD, CAT), and modulated neuroprotective gene expressions (BDNF, CREB1, Nrf2). Both crude plant extracts and isolated flavonoids, administered primarily via oral routes, demonstrated robust efficacy. Animal models included Swiss albino mice, Wistar rats, and zebrafish.

**Conclusions:**

Flavonoids exhibit multi-targeted neuroprotection against amnesia by enhancing synaptic plasticity, combating oxidative damage, and modulating apoptotic pathways. These findings support advancing flavonoids toward clinical applications for cognitive disorders, warranting further translational studies and optimized formulations.

**List of abbreviations**
AChE: Acetylcholinesterase
AMPK: AMP-activated Protein Kinase
Akt: Protein Kinase B
APP/PS1: Amyloid Precursor Protein/Presenilin-1 (transgenic mouse model)
BDNF: Brain-Derived Neurotrophic Factor
CAT: Catalase
CREB: cAMP Response Element-Binding Protein
ERK: Extracellular Signal-Regulated Kinase
FOXO-1: Forkhead Box O1
GSH: Glutathione
LPO: Lipid Peroxidation
LTP: Long-Term Potentiation
MDA: Malondialdehyde
mTOR: Mammalian Target of Rapamycin
MWM: Morris Water Maze
NF-κB: Nuclear Factor Kappa-Light-Chain-Enhancer of Activated B Cells
NORT: Novel Object Recognition Test
NPY: Neuropeptide Y
Nrf2: Nuclear Factor Erythroid 2–Related Factor 2
PTEN: Phosphatase and Tensin Homolog
ROS: Reactive Oxygen Species
SIRT1: Silent Information Regulator 1
SOD: Superoxide Dismutase
YMT: Y-Maze Test

## Introduction

Amnesia, broadly defined as the loss or disruption of memory function, represents a major clinical challenge with profound personal, social, and economic consequences. ^1^ It can arise from diverse etiologies, including neurodegenerative diseases such as Alzheimer’s disease, traumatic brain injury, cerebrovascular events, infections, and psychiatric conditions. Despite the underlying causes varying significantly, a common feature across many forms of amnesia is neuronal dysfunction or death within brain regions critical for memory processing, particularly the hippocampus and associated cortical structures. ^2^ Current pharmacological therapies, including cholinesterase inhibitors and NMDA receptor antagonists, offer limited efficacy and are often associated with undesirable side effects. This therapeutic gap has intensified interest in exploring safer, more effective neuroprotective agents, particularly those derived from natural sources. ^3^

Flavonoids, a large group of polyphenolic compounds naturally occurring in fruits, vegetables, teas, and medicinal plants, have gained considerable attention due to their diverse pharmacological activities. ^4^ Numerous experimental studies have demonstrated that flavonoids exert antioxidant, anti-inflammatory, anti-apoptotic, and neurotrophic effects, all of which are mechanisms critically implicated in memory preservation and cognitive enhancement. Flavonoids are known to modulate critical signaling pathways, including ERK/CREB/BDNF and PI3K/Akt, which are central to synaptic plasticity, neurogenesis, and long-term potentiation, the molecular substrates of memory formation and retrieval. ^5^ Additionally, flavonoids have been observed to attenuate oxidative stress-induced neuronal damage and inhibit neuroinflammation, both of which are prominent pathological features in various models of amnesia. ^6^ Despite the biological plausibility and encouraging preclinical findings, the evidence surrounding the protective effects of flavonoids in amnesia has yet to be systematically synthesized. The diversity of flavonoid subclasses, varying models of amnesia, and different outcome measures employed across studies make it necessary to critically appraise and integrate available data. Such a synthesis will help identify the most promising flavonoid compounds, clarify their mechanisms of action, and guide future research toward the development of flavonoid-based therapeutic interventions for memory disorders. ^7^

Therefore, the primary objective of this systematic review is to evaluate existing experimental evidence on the protective role of flavonoids in amnesia. Specifically, this review will focus on assessing the efficacy of different flavonoid compounds, elucidating their mechanisms of neuroprotection, and identifying key knowledge gaps that warrant further investigation.

## Materials and methods

### Study design

This systematic review was designed to comprehensively investigate the protective role of flavonoids in amnesia. The review adhered strictly to the Preferred Reporting Items for Systematic Reviews and Meta-Analyses (PRISMA) 2020 guidelines to ensure transparency, accuracy, and reproducibility throughout the process.

### Literature search strategy

A comprehensive literature search of published articles investigating the protective role of flavonoids in amnesia was conducted across three major electronic databases: Web of Science (WoS), Scopus, and PubMed. The search was performed on the 10 ^th^ of December 2024. Search terms included the keywords “flavonoid” and “amnesia,” with the search fields restricted to “Title,” “Abstract,” and “Keywords” in WoS and Scopus databases to ensure a robust and targeted retrieval of relevant studies. Boolean operators (AND, OR, NOT), quotation marks, parentheses, wildcards, and asterisk (*) symbols were strategically employed to optimize the search results, following established search optimization techniques. The search strategy combined multiple related flavonoid terms and was structured as follows for WoS and Scopus: (“flavonoid” OR “flavonol” OR “flavanone” OR “flavone” OR “flavan-3-ol” OR “isoflavone” OR “daidzein” OR “genistein” OR “kaempferol” OR “apigenin” OR “catechin” OR “epicatechin” OR “epigallocatechin” OR “gallocatechin” OR “luteolin” OR “hesperetin” OR “quercetin” OR “biochanin” OR “theaflavin” OR “formononetin” OR “baicalein” OR “myricetin” OR “chrysin” OR “naringenin” OR “glycitein” OR “eriodictyol” OR “isorhamnetin” OR “thearubigin” OR “anthocyanin” OR “delphinidin” OR “peonidin” OR “malvidin” OR “anthocyanidin” OR “petunidin” OR “cyanidin” OR “pelargonidin”) AND (“amnesia”). For PubMed, the search was adapted to include MeSH terms and field tags, resulting in the following syntax: (“flavonoid” [Title/Abstract] OR “flavonol” [Title/Abstract] OR …) AND (“amnesia” [Title/Abstract]) NOT (review [Publication Type]).

### Screening and selection process

All retrieved articles from the database searches were downloaded in CSV format and subsequently uploaded onto the Rayyan platform, a web-based tool designed to streamline systematic review processes. Rayyan was used to facilitate the efficient and blinded screening of titles and abstracts based on predefined eligibility criteria. Duplicate entries were automatically identified and removed by the Rayyan system before the screening commenced. The selection process followed three sequential stages: title screening, abstract screening, and full-text review. Each article was assessed for relevance against the established inclusion and exclusion criteria and any disagreements during screening were resolved through discussion and consensus among the reviewers. Screening and selection were conducted in strict adherence to the Preferred Reporting Items for Systematic Reviews and Meta-Analyses (PRISMA) 2020 guidelines. Only studies that met all eligibility criteria at each screening level were included for final qualitative synthesis. Articles that failed to meet the criteria were excluded at the appropriate stage, with exclusion reasons documented systematically. Two independent reviewers screened and extracted data from each included article using a predefined data extraction form. Disagreements were resolved through discussion, and no automation tools were used in the data collection process.

### Inclusion and exclusion criteria

Studies that investigated the role of flavonoids in amnesia, including those that evaluated any flavonoid compound for the treatment or management of amnesia, were included. Eligible studies specifically targeted flavonoid-based interventions and reported on the protective functions or therapeutic roles of flavonoids in amnesia. Only studies that presented quantitative outcomes related to the severity of amnesia, were published in peer-reviewed journals in English, and demonstrated clear methodology with detailed reporting regarding the administration of flavonoids or formulations, compounds, or extracts with flavonoid activity were considered for inclusion. Studies that did not focus on flavonoids, lacked experimental models of amnesia (with clinical trials also excluded), or investigated therapies unrelated to flavonoid interventions were excluded. Additionally, studies published in languages other than English, studies without clear reporting of outcomes or methodology, and secondary sources such as review articles, editorials, commentaries, and conference abstracts were excluded from the review.

### Data synthesis and analysis

Extracted data were tabulated and categorized under distinct headings for clarity. Descriptive synthesis was employed to summarize the outcomes, focusing on experimental models, types of flavonoids, behavioral assessments, biochemical markers, gene expression, and affected brain regions. Frequencies and percentages were calculated for key variables, and where possible, trends and correlations were discussed. The Rayyan platform facilitated structured data management throughout the analysis process.

## Results

### Search results

**Figure 1.**
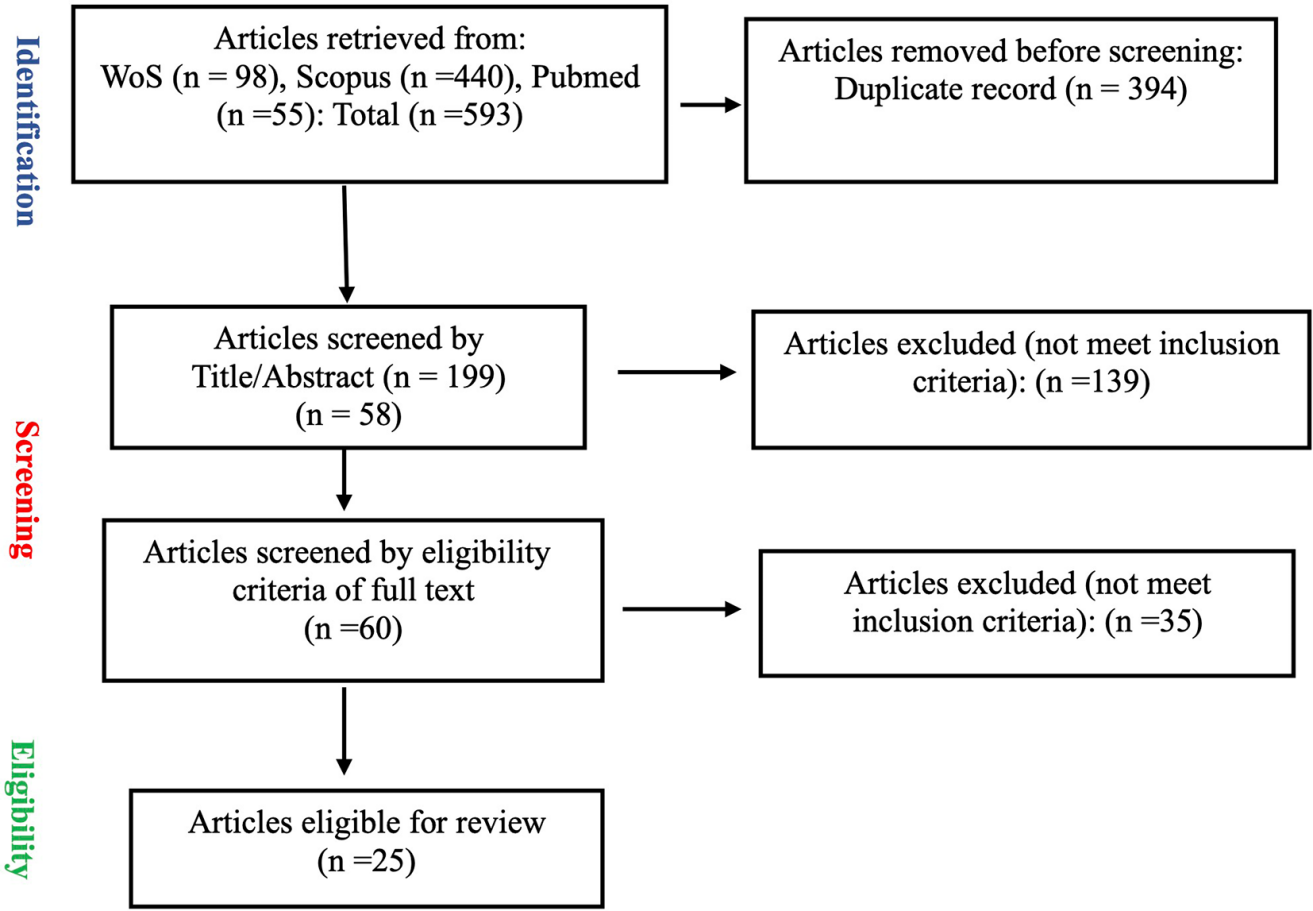
PRISMA study selection flow chart.

A total of 593 articles were retrieved from three databases: 98 from Web of Science, 440 from Scopus, and 55 from PubMed. After the removal of 394 duplicates, 199 articles proceeded to the screening stage. Screening of titles and abstracts resulted in the exclusion of 139 articles. The full texts of the remaining 60 articles were then assessed for eligibility, leading to the exclusion of 35 articles that did not meet the inclusion criteria. In the end, 25 articles were included in the final review after completing the eligibility and quality assessment process as depicted in Figure 1.

### Study characteristics

These studies were conducted across various countries, demonstrating considerable diversity in animal models, species, sample sizes, sexes, and age groups used in the research. The studies spanned multiple countries, including Brazil, Pakistan, Cameroon, India, China, Saudi Arabia, South Korea, Tunisia, Romania, Egypt, the United Arab Emirates, and Mongolia, along with contributions from unspecified locations. A variety of animal models were employed, with rats and mice being the most commonly used. Zebrafish and gerbils were also utilized in several studies. Among the mouse models, specific strains included Swiss albino mice, Laca mice, and transgenic APP/PS1 mice. For rat models, Wistar and Sprague-Dawley strains were frequently used. The majority of studies predominantly used male animals, though some included both sexes. Sample sizes varied widely, ranging from as few as six animals per group to larger cohorts of over 100 animals. Animal ages were also diverse, with studies reporting precise age ranges or weights. For instance, some studies focused on young adult animals, while others used aged animals to model disease progression. Several studies also detailed treatment initiation and assessment at specific age milestones. Full details of species, strains, sex distribution, sample sizes, and ages are summarized in Table 1.

**Table 1.** Study Characteristics.

S/N	Reference	Country	Animal species	Animal type	Gender of animals	Sample size	Age of animals (wks)
1	^8^	Brazil	Swiss Mice	Mice	Male	82	8 wks
2	^9^	Pakistan	Sprague-Dawley	Rats	Male	30	
3	^10^	India	Mice	Swiss mice	NA	NA	NA
4	^11^	Cameroon	Mice	Swiss albino mice	Both sexes	35 mice	2-2.5 months old
5	^12^	NA	Mice	Swiss albino mice	NA	NA	NA
6	^13^	NA	Rats	NA	NA	NA	NA
7	^14^	China	Rats	Sprague Dawley rats	Female	60 rats	6 months old
8	^15^	NA	Rats	Wistar rats	Males	40 rats	60 days old
9	^16^	NA	Mice	APP/PS1 transgenic mice and wild-type mice	NA	NA	Treatment at 5 months assay at 7 months
10	^17^	South Korea	Gerbils	Mongolian gerbils	Male	106 gerbils (varied across groups)	Six months old
11	^18^	NA	Mice	NA	NA	NA	NA
12	^7^	NA	Rats	Sprague-Dawley rats	Sprague-Dawley rats	NA	NA
13	^19^	NA	Rats	Wistar rats	Male	12 rats per group	Adult
14	^6^	NA	Mice	Institute of Cancer Research (ICR) mice	NA	NA	4 weeks old
15	^20^	NA	Mice	Swiss albino mice	NA	NA	NA
16	^20^	NA	Rats	NA	NA	NA	NA
17	^5^	NA	Zebrafish	Danio rerio	NA	NA	NA
18	^21^	NA	Mice	NA	NA	NA	Adult
19	^22^	Tunisia, Romania, Egypt, United Arab Emirates	Zebrafish	Danio rerio	NA	Eight animals per group	NA
20	^23^	India	Rats	Wistar rats	Male	6 rats per group	12 months old
21	^24^	Pakistan	Mice	Swiss albino mice	Male	144 mice	25-30 g body weight
22	^25^	NA	Mice	Laca mice	Male	6 mice per group	3-4 months old
23	^26^	NA	Mice	Swiss albino mice	NA	NA	NA
24	^27^	Iran	Rats	Male rats	Male	NA	NA
25	^28^	Mongolia	Mice	NA	NA	NA	NA

The studies reviewed involved a variety of animal models, predominantly Swiss albino mice, Wistar rats, and Sprague-Dawley rats, with occasional use of zebrafish (Danio rerio) and Mongolian gerbils. The majority of studies used male animals, though some included both sexes or did not specify gender. Sample sizes varied considerably, with most studies using between 6 to 60 animals per group. Animal ages ranged from young adults (2 months) to aged models, with several studies initiating treatment around 4 to 12 weeks of age. These animal models and demographic variations were selected to closely mimic neurodegenerative and cognitive impairment conditions for evaluating the neuroprotective and antioxidant effects of plant extracts and flavonoid.

### Behavioral tests and animal models utilized

**Figure 2.**
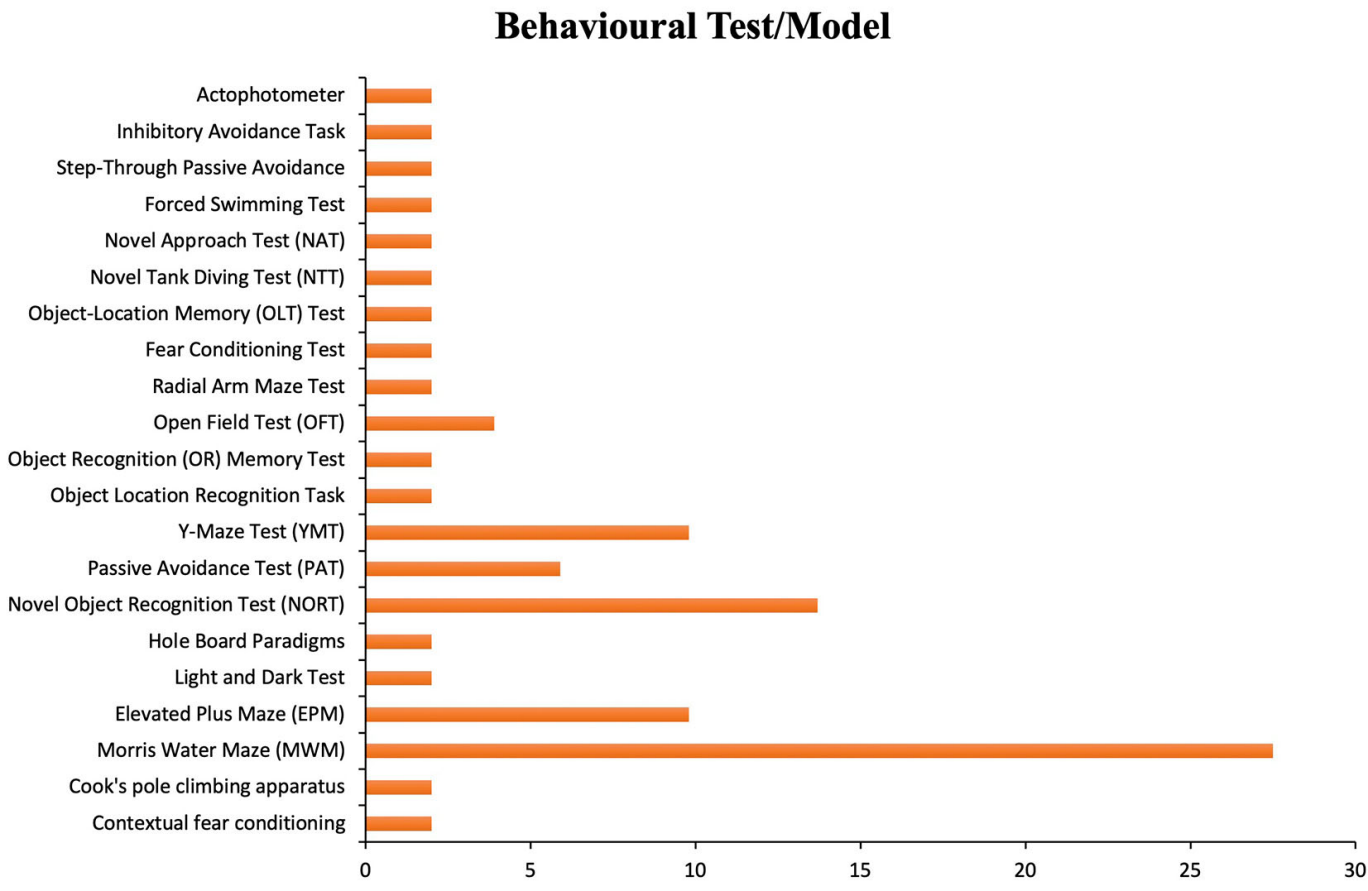
Frequency of behavioral tests used in flavonoid-amnesia studies.

The analysis of behavioral models employed across the reviewed studies demonstrates a clear preference for specific memory and learning assessment paradigms as depicted by Figure 2. The Morris Water Maze (MWM) was the most frequently utilized test, reflecting its robust sensitivity for evaluating spatial learning and memory functions, particularly hippocampus-dependent processes. ^25^ The Novel Object Recognition Test (NORT) and Y-Maze Test (YMT) also featured prominently, emphasizing the importance of assessing recognition memory and spontaneous alternation behavior as measures of cognitive flexibility. ^30^ Other commonly used models included the Elevated Plus Maze (EPM) and Passive Avoidance Test (PAT), suggesting a broader interest in anxiety-related memory retention and avoidance learning. Less frequently employed tests, such as the Radial Arm Maze and Object-Location Memory Test, provide complementary assessments of working memory and object-place associations. Overall, the distribution of behavioral models indicates that researchers prioritized multi-dimensional evaluation of memory, integrating spatial, recognition, and emotional memory paradigms to capture the complexity of amnesia-related cognitive deficits.

### Genes expressed

**Oxidative stress markers assessed**

**Figure 3.**
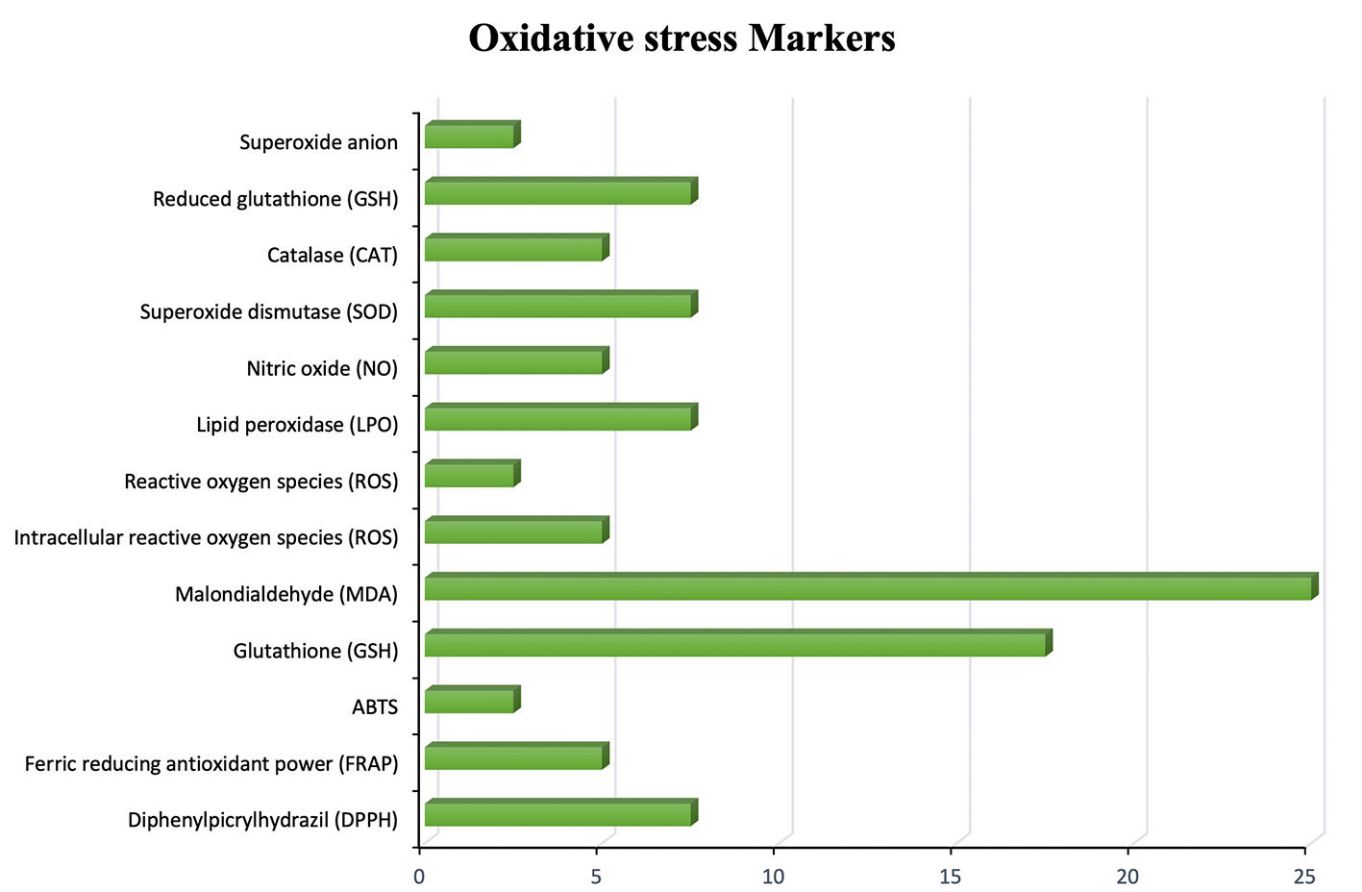
Frequency of oxidative stress markers measured in flavonoid-amnesia studies.

The evaluation of oxidative stress markers across the included studies highlights the critical role of oxidative damage in amnesia and the potential antioxidative benefits of flavonoid interventions as depicted by Figure 3. Malondialdehyde (MDA) was the most frequently measured marker, reflecting its well-established role as a sensitive indicator of lipid peroxidation and cellular oxidative injury. ^31, 32^ Glutathione (GSH) and superoxide dismutase (SOD) were also commonly assessed, underscoring the importance of endogenous antioxidant defenses in maintaining neuronal integrity. ^33^ Other markers such as lipid peroxidase (LPO), catalase (CAT), and nitric oxide (NO) were frequently utilized, further emphasizing the multifactorial nature of oxidative damage pathways in cognitive dysfunction. ^34^ Less frequently assessed indicators, including FRAP, DPPH, and reactive oxygen species (ROS), provided complementary measures of overall antioxidant capacity and free radical scavenging activity. ^35^ Overall, the pattern of biomarker evaluation supports that oxidative stress modulation is a central mechanism through which flavonoids exert neuroprotective effects against amnesia.

**Plant extracts, phytochemicals, dosages, and routes of administration**

The reviewed studies demonstrate the diverse use of plant extracts and isolated flavonoid compounds in the experimental management of amnesia, as summarised in Table 3. Oral administration emerged as the predominant route, reflecting its practicality and relevance for translational research into human therapies. Most studies utilized ethanolic or methanolic extracts (e.g., *Averrhoa carambola*, *Grewia asiatica*, *Cyamopsis tetragonoloba*), highlighting the solubility of active flavonoids in polar solvents and the richness of polyphenolic compounds in these preparations. ^10, 9, 12^ Doses ranged widely from low concentrations (e.g., 5 mg/kg for Biochanin A) to relatively high doses (e.g., 800 mg/kg for *Lavandula stoechas* extract), suggesting differences in extract potency, bioavailability, and experimental design across studies. ^18^ Standardized compounds such as quercetin, kaempferol, silibinin, and pycnogenol® were also assessed, providing insights into the neuroprotective potential of purified flavonoids. ^7, 17, 19^ Less common administration methods, such as intraperitoneal injection ( *Pinus pinaster*, *Glycine max*) and immersion ( *Tribulus terrestris*), highlight methodological adaptations for certain experimental models. Notably, several studies did not report dosage or administration details, which may affect reproducibility and interpretation. ^17^ Overall, the data suggest that both crude extracts and purified flavonoid compounds confer neuroprotective effects against amnesia through varied dosing strategies and predominantly oral delivery, supporting their therapeutic potential.

**Table 2.** Genes expressed from studies.

S/N	Gene expression	Organ	Reference
1	AChE and ChAT enzyme gene expression	N/A	^8^
2	Bcl-2, Bax, Caspase-3	Hippocampus	^14^
3	bdnf, npy, egr-1, nfr2α, creb1	Brain	^5^
4	ERK and beta-actin proteins	Hippocampus	^29^
5	Extracellular signal-regulated kinase (ERK), cAMP response element-binding protein (CREB), BDNF	Hippocampus	^6^
6	Silent information regulator 1 (SIRT1), Nuclear factor kappa B (NF-κB), p53, Forkhead box O (FOXO-1)	Hippocampus	^19^
7	Immediate early gene cFos, mitochondrial dynamic markers (Dynamin-related protein-1, Mitofusins 1 and 2)	Hippocampus	^27^
8	Nrf2, Bcl2, Bax, cleaved caspase-3	Hippocampus	^19^

**Table 3.** Plant extracts, phytochemicals, dosages, and routes of administration from various studies.

S/N	Plant name	Extract/phytochemicals used	Dosage of administration	Route of administration	Reference
1	*Averrhoa carambola*	Ethanolic extract	100 mg/kg, 200 mg/kg, and 400 mg/kg	Oral	^10^
2	Baicalein ( *Scutellaria baicalensis*)	Flavonoid (baicalein)	NA	Oral (via drinking water)	^20^
3	*Biochanin A* (Red clover, cabbage)	Biochanin A (BCA)	5 mg/kg, 20 mg/kg, and 60 mg/kg	Oral gavage	^14^
4	Blackcurrant ( *Ribes nigrum* L.)	NA	50 mg/kg	Oral	^8^
5	*Cajanus cajan*	Ethanol seed extract	50, 100, or 200 mg/kg	Oral	^21^
6	*Capparis sepiaria*	Aqueous lyophilisate	10 mg/kg and 40 mg/kg	Oral gavage	^11^
7	*Carthamus tinctorius* L.	Kaempferol	50 mg/kg	Oral	^19^
8	*Citrus limon*	Juice	0.6 ml/kg/day and 1.2 ml/kg/day	Oral	^3^
9	*Cyamopsis tetragonoloba*	Methanolic tender green pod extract	100 mg/kg and 200 mg/kg	Oral	^12^
10	*Dimorphandra mollis*	Flavonoids	NA	NA	^8^
11	*Elaeagnus umbellate*	Methanolic extract	200 mg/kg (CHF Ext), 30 mg/kg (Chlorogenic)	Oral	^24^
12	French maritime pine ( *Pinus pinaster*)	Pycnogenol®	30, 40, and 50 mg/kg	Intraperitoneal injection	^17^
13	Glycine max (soybean)	Textured soy protein (TSP) extract	100 mg/kg, 200 mg/kg, and 400 mg/kg	Intraperitoneal (i.p.)	^27^
14	*Grewia asiatica* L.	Quercetin, Anthocyanin	200 mg/kg	Oral	^9^
15	*Lactuca sativa* (LS)	Ethanol extract of fresh LS leaves (LSEE)	50, 100, and 200 mg/kg (LSEE); 15 mg/kg (n-Bu)	Oral	^36^
16	*Lavandula stoechas*	Methanolic extract	800 mg/kg	Oral	^18^
17	*Lawsonia inermis*	Leaves extract	NA	NA	^20^
18	*Limonia acidissima*	Methanolic extract	NA	NA	^13^
19	*Manihot esculenta* Crantz	Cassava leaves' extract	NA	Oral	^15^
20	Milk thistle	Silibinin (silybin)	NA	NA	^16^
21	Ocimum species	Hydro-methanol extract	200 mg/kg and 400 mg/kg	Oral	^31^
22	Onion (Quergold cultivar)	Quercetin	NA	Oral	^23^
23	*Sesbania grandiflora*	Ethanolic extract	NA	Oral	^37^
24	Soybean	Soy isoflavones	40 mg/kg	Oral	^6^
25	Tribulus terrestris	Ethanolic extract	1, 3, and 6 mg/L	Immersion	^22^

**Animal models utilized**

**Figure 4.**
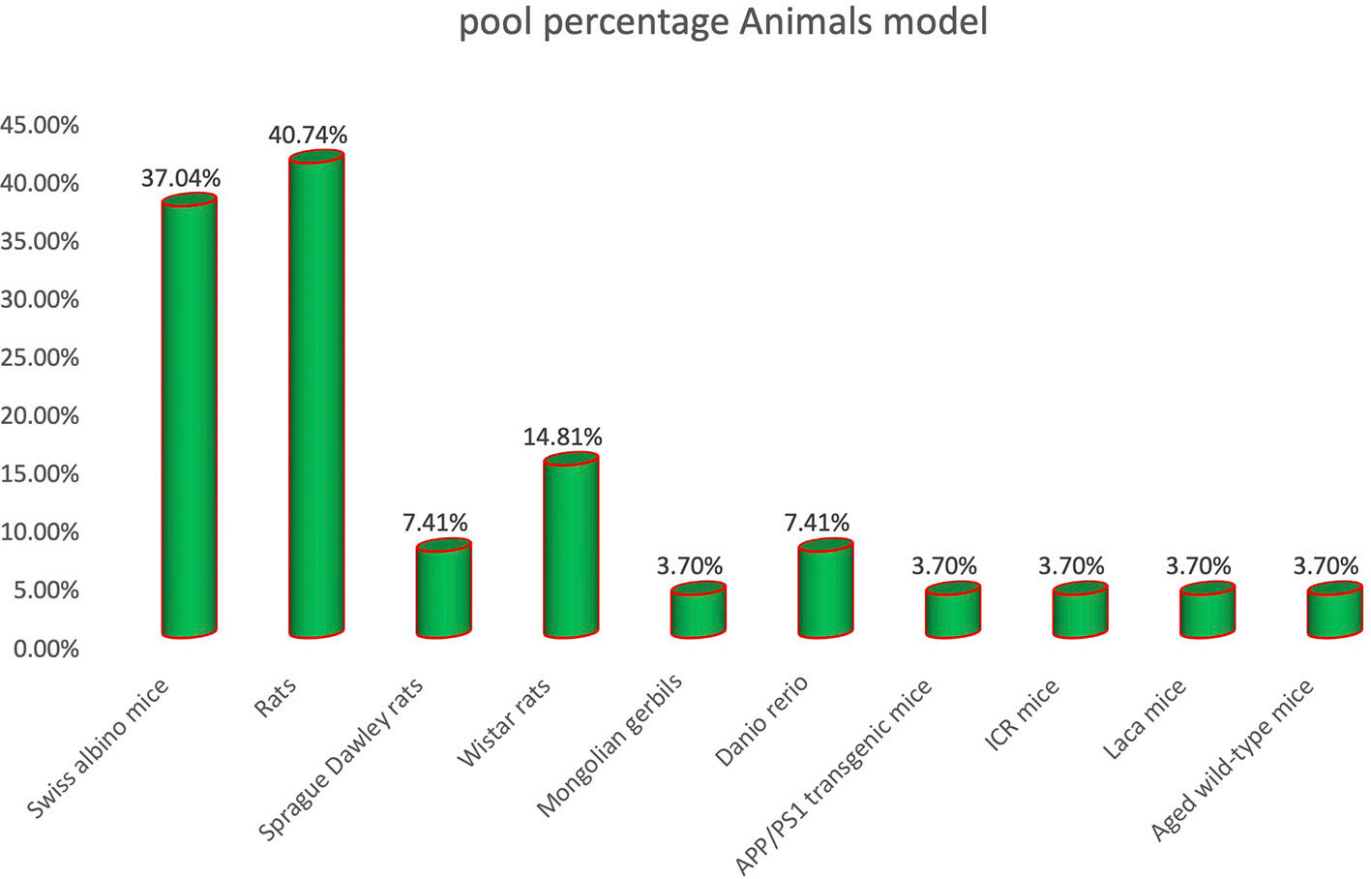
Distribution of animal models used in studies investigating flavonoid effects on amnesia.

The distribution of animal models in the reviewed studies highlights a predominant reliance on rats (40.74%) and Swiss albino mice (37.04%) for evaluating the protective effects of flavonoids against amnesia, as depicted in Figure 4. This preference reflects the extensive validation of these models in behavioral neuroscience, particularly in memory and learning paradigms. Among rat models, Wistar rats (14.81%) and Sprague Dawley rats (7.41%) were commonly employed due to their well-characterized cognitive profiles and reproducibility in experimental settings. ^29, 30^ The use of Danio rerio (zebrafish) (7.41%) illustrates emerging trends favoring alternative, cost-effective models for high-throughput behavioral screening. ^21, 22^ Less frequently used models, including Mongolian gerbils, APP/PS1 transgenic mice, ICR mice, Laca mice, and aged wild-type mice (each at 3.70%), reflect specific study designs targeting particular aspects of neurodegeneration, aging, or genetic predisposition to memory deficits. Overall, the data indicate a strong preference for traditional rodent models, with growing exploration of alternative species to diversify cognitive research in amnesia.

## Discussion

This systematic review critically evaluated the neuroprotective potential of flavonoids against amnesia, integrating findings from behavioral assessments, oxidative stress biomarkers, gene expression studies, plant-based interventions, and animal models. Overall, the evidence consistently supports that flavonoids exert multi-targeted therapeutic actions, highlighting their promise as candidates for managing cognitive impairment.

Behavioral assessments revealed that the Morris Water Maze (MWM) was the most widely employed model, emphasizing its sensitivity to hippocampus-dependent spatial memory loss. This observation aligns with prior studies ^25, 30^ Which have established MWM as a gold standard in memory research. Additionally, the frequent use of the Novel Object Recognition Test (NORT) and Y-Maze Test (YMT) reflects a growing trend toward adopting multidimensional cognitive testing that captures both spatial and recognition memory domains. ^24^ Compared to older reviews that emphasized single-task paradigms, the diversification seen here suggest a methodological advancement.

In parallel with behavioral outcomes, biochemical findings consistently demonstrated that oxidative stress plays a central role in amnesia pathogenesis. Malondialdehyde (MDA) was the most frequently measured biomarker, reinforcing its established position as an indicator of lipid peroxidation and neuronal oxidative injury. ^38^ Alongside MDA, antioxidant enzymes such as glutathione (GSH), superoxide dismutase (SOD), and catalase (CAT) were commonly evaluated, highlighting the importance of maintaining redox balance in memory preservation. Compared to earlier studies that mainly focused on MDA as a damage marker, the broader antioxidant profiling observed here indicates a more complex understanding of oxidative dynamics. ^39^ These findings imply that flavonoids’ ability to simultaneously suppress oxidative stress and enhance antioxidant defenses could offer a dual-layered strategy superior to conventional single-mechanism antioxidants. ^2^

Molecular evidence from gene expression studies as depicted by Table 2 further strengthened these observations. Flavonoid interventions upregulated key neurotrophic and memory-related genes, notably BDNF, CREB1, and NPY, while downregulating pro-apoptotic markers such as Bax and cleaved caspase-3. This dual modulation of neuroplasticity and survival pathways reflects a complex neuroprotective mechanism, consistent with findings reported by Ref. 3. In contrast to earlier polyphenol studies that focused narrowly on BDNF activation, the broader pathway engagement observed here suggests that flavonoids may offer more robust synaptic resilience. ^4^ This suggests that the ability of flavonoids to target multiple molecular pathways simultaneously makes them particularly advantageous for treating complex neurodegenerative conditions like Alzheimer’s disease, where several mechanisms of cognitive decline occur together. ^40^

The diversity of plant-derived interventions is summarized in Table 4, which presents the key findings and conclusions from studies involving flavonoid-rich extracts and compounds. The reviewed studies consistently demonstrated that flavonoids improved memory functions, enhanced antioxidant defenses, modulated apoptosis, and supported cholinergic neurotransmission. ^21, 30, 39^ Some compounds, such as biochanin A, baicalein, kaempferol, and soy isoflavones, showed effects comparable to conventional anti-amnesic agents like donepezil, suggesting that flavonoids can offer similar therapeutic efficacy with fewer side effects. ^12, 26^ Moreover, several studies such also demonstrated synergistic effects when flavonoids were combined with standard therapies, indicating potential as adjunct treatments. ^41^

**Table 4.** Key findings and conclusions from the included studies.

S/N	Reference	Key findings/outcomes	Conclusion
1	^8^	Blackcurrant improved memory and oxidative balance by cholinergic modulation.	Promising natural supplement for memory impairment.
2	^9^	G. asiatica flavonoids boosted acetylcholine, reduced stress, enhanced memory.	Effective for neuroprotection and cognitive recovery.
3	^10^	Naringenin improved memory and redox status in cadmium-treated mice.	Protects against cadmium-induced neurotoxicity.
4	^11^	C. sepiaria enhanced brain antioxidants and reduced AChE.	Shows anti-amnesic potential via antioxidant and cholinergic effects.
5	^12^	Cyamopsis extract reversed memory loss like donepezil.	Multi-target cognitive impairment therapy candidate.
6	^13^	Limonia extract enhanced behavior, decreased AChE, improved brain histology.	Enhances memory in Alzheimer’s models.
7	^14^	Biochanin A improved cognition, redox balance, and apoptosis markers.	Neuroprotective for postmenopausal cognitive decline.
8	^15^	Cassava leaves extract preserved memory and antioxidant levels in the hippocampus.	Preventive potential in Alzheimer’s disease.
9	^15^	Baicalein restored LTP, reduced Aβ, tau, and synaptic deficits.	Promising oral therapy for Alzheimer’s.
10	^17^	Pycnogenol® protected memory and neurons from ischemic damage via antioxidants.	Effective post-stroke neuroprotectant.
11	^18^	Lavandula stoechas improved memory and increased SOD, CAT, GSH.	Stabilizes memory via cholinergic and antioxidant support.
12	^7^	Silibinin reduced inflammation, improved autophagy, and prevented memory loss.	Promising for Alzheimer’s treatment.
13	^19^	Kaempferol improved behavior, antioxidant markers, and signaling regulation.	Neuroprotection through PTEN, AMPK, Akt/mTOR pathway.
14	^6^	Soy isoflavones enhanced cognition, reduced stress, upregulated BDNF, ERK, CREB.	Powerful neuroprotectant in memory dysfunction.
15	^20^	Lawsonia inermis enhanced cholinergic activity and antioxidant defenses.	Boosts memory via neurotransmission and redox pathways.
16	^20^	Baicalin reduced oxidative stress, apoptosis, and improved learning.	Potential agent for Alzheimer’s disease.
17	^5^	Rhoifolin improved memory and increased gene expression in zebrafish.	Promising for amnesia and anxiety treatment.
18	^21^	Cajanus cajan enhanced working memory and antioxidant enzymes, lowered AChE.	Improves cognition via cholinergic and antioxidant effects.
19	^22^	Tribulus terrestris showed anxiolytic effects via MAO-A interaction.	Herb has antidepressant and anxiolytic potential.
20	^23^	Quercetin + donepezil improved cognition, reduced AChE and oxidative stress.	Combined therapy enhances anti-amnesic effects.
21	^24^	Elaeagnus umbellata improved spatial memory and novel object recognition.	Chlorogenic acid is an effective anti-amnesic compound.
22	^25^	Lactuca sativa reversed memory loss in scopolamine models.	Validates its traditional use as memory enhancer.
23	^26^	Ocimum basilicum reduced AChE, boosted antioxidants, and improved memory.	Neuroprotective and anti-amnesic herb.
24	^27^	TSP extract improved learning and antioxidant defense.	Enhances memory via isoflavone-based pathways.
25	^28^	RDP decreased ROS, prevented apoptosis, and activated BDNF/TrkB pathway.	Antioxidant-rich supplement for neurodegenerative disorders.

Furthermore, plant-based interventions showed substantial diversity, including both crude extracts and isolated flavonoids such as quercetin, kaempferol, baicalein, and silibinin. ^8, 9, 42^ Oral administration was the predominant route, consistent with natural dietary intake and supporting pharmacokinetic evidence that flavonoids exhibit moderate to good oral bioavailability. ^1^ Although a few studies used intraperitoneal or immersion methods, these alternative routes may limit clinical translation. Compared to earlier flavonoid studies that often-lacked route standardization, the predominance of oral administration here reflects a growing focus on translational relevance. It implies that optimizing oral delivery systems, including nano-formulations or bioenhancers, should be a future research priority to maximize therapeutic effectiveness.

Animal model analysis demonstrated a strong preference for traditional rodents, with rats (40.74%) and Swiss albino mice (37.04%) most commonly used. This mirrors established trends in cognitive neuroscience due to the reliability and depth of behavioral characterization available for these models. However, the emerging use of zebrafish (Danio rerio) and transgenic APP/PS1 mice suggests a shift toward models that better represent aging, genetic vulnerability, and high-throughput capabilities. In contrast to older research that relied almost exclusively on rodents, the diversification observed here reflects an important methodological evolution. The implication is that using a broader range of models can improve the generalizability and translational relevance of preclinical flavonoid research. ^43^

Additionally, the cumulative outcomes from the reviewed studies confirm that flavonoids improve cognitive performance, enhance antioxidant defenses, modulate key neurotrophic and apoptotic signaling pathways, and preserve cholinergic function. ^18, 19^ Compared to traditional antioxidant strategies or single-target drugs, flavonoids appear to operate across multiple pathological processes simultaneously. This observation aligns with broader polyphenol research and highlights the potential of flavonoids to offer superior, multi-faceted interventions for memory disorders. ^44^ Importantly, the inclusion of studies demonstrating synergistic effects between flavonoids and standard therapies (e.g., quercetin combined with donepezil) suggests that flavonoids could complement existing treatments rather than replace them, providing an additive benefit.

## Conclusion

This systematic review provides strong evidence that flavonoids possess significant neuroprotective properties against amnesia through multi-targeted mechanisms. The reviewed studies consistently demonstrated that flavonoid interventions improve cognitive performance by reducing oxidative stress, enhancing antioxidant defenses, promoting synaptic plasticity, and regulating apoptotic pathways. Both crude plant extracts and isolated flavonoid compounds showed remarkable efficacy across various animal models, predominantly through oral administration routes, suggesting their translational potential for human therapy. The modulation of key molecular pathways, including upregulation of BDNF, CREB1, and Nrf2, highlights flavonoids’ ability to support neurogenesis and cognitive resilience. These findings are consistent with broader polyphenol research and emphasize that flavonoids offer a holistic approach to mitigating cognitive decline, superior to single-target interventions. Despite encouraging results, substantial heterogeneity exists in experimental designs, dosages, and extract standardization. Future research should focus on harmonizing methodologies, enhancing bioavailability, and conducting rigorous clinical trials to validate these promising preclinical outcomes. The integration of flavonoids into clinical practice could offer a safe, accessible, and effective strategy for preventing and managing amnesia and related neurodegenerative conditions, especially in aging populations. Overall, flavonoids represent a promising class of natural compounds with the potential to significantly advance cognitive health interventions.

### Limitations

This systematic review is limited by its exclusive focus on preclinical experimental studies conducted in animal models, which restricts the generalizability of the findings to human populations. The lack of clinical trials highlights a translational gap that must be addressed to confirm these results in clinical settings. There was considerable heterogeneity across the included studies in terms of animal species, flavonoid types, dosages, routes of administration, and behavioral assessment tools. This variability hindered direct comparisons across studies and made meta-analysis infeasible. Many studies did not provide detailed phytochemical characterization or standardization of the flavonoid content in their extracts. This absence of consistency affects the reproducibility and pharmacological interpretation of the findings. In several cases, the duration of treatment and follow-up periods was either unclear or not reported, limiting conclusions about the sustainability of effects over time. Risk of bias assessments and certainty grading of evidence, such as the use of GRADE, were not conducted, which weakens the ability to critically appraise the overall strength of the evidence. The review was also limited to studies published in English, which may have introduced language bias and excluded potentially relevant findings from non-English sources. These limitations emphasize the importance of methodological standardization, clinical translation, and broader inclusion criteria in future systematic reviews on this topic.

### Recommendations

Future research should prioritize the standardization of flavonoid preparations and dosing protocols to enhance comparability. Rigorous pharmacokinetic and pharmacodynamic studies are necessary to optimize bioavailability and therapeutic windows. To enhance clinical relevance, translational studies, including well-designed human trials, should be conducted to validate the neuroprotective efficacy of flavonoids in amnesia and related cognitive disorders. Combining flavonoids with current pharmacotherapies should also be explored for potential synergistic effects. Additionally, the development of flavonoid-based nutraceuticals or supplements with well-defined compositions and safety profiles may offer accessible interventions, especially in aging or at-risk populations. Finally, future reviews should consider broader inclusion criteria, such as non-English studies and grey literature, to capture a more holistic evidence base.

## Disclosure statement

The authors declare that the content of this systematic review is solely the result of their independent academic work. No external influence or conflict affected the integrity of the data interpretation, synthesis, or reporting.

## Ethical approval

This study is based exclusively on the synthesis of previously published preclinical studies and did not involve any direct research with human participants or animals conducted by the authors. Therefore, ethical approval and participant consent were not required.

## Data Availability

No data associated with this article. PRSIMA: THE PROTECTIVE ROLE OF FLAVONOIDS IN AMNESIA -SYSTEMATIC REVIEW, DOI: 10.6084/m9.figshare.29351495.v1 ^45^ This dataset is available under the terms of the Creative Commons Attribution 4.0 International license (CC BY 4.0), which permits unrestricted use, distribution, and reproduction in any medium, provided the original work is properly cited.
