# Supplementary material for: THE PROTECTIVE ROLE OF FLAVONOIDS IN AMNESIA -SYSTEMATIC REVIEW
Source: F1000Res. 2025 Jul 21;14:716. [Version 1] doi: 10.12688/f1000research.164798.1 (PMC12442874; doi:10.12688/f1000research.164798.1)
Supplement: Peer review history

# Reviewer report: Reviewer response for version 1

**DOI:** 10.5256/f1000research.181373.r408295

**Published:** 2025-09-16

**Authors:** Yujun Hou

**Affiliations:** 1 Tongji University, Shanghai, Shanghai, China

The authors present a PRISMA-compliant systematic review of pre-clinical studies (2010–2024) investigating flavonoid-based interventions in animal models of amnesia. Out of 593 screened records, 25 rodent and zebrafish studies were included. The review highlights consistent improvements in behavioral performance, reductions in oxidative stress markers, and modulation of neuroplasticity and apoptosis-related genes. Based on these findings, the authors suggest that flavonoids represent promising multi-target neuroprotectants with translational potential. While the topic is of high relevance and the synthesis provides useful insights, there are several methodological and reporting issues that must be addressed to improve the rigor, clarity, and overall impact of the work.

**Major Comments**

1. Table 1 contains numerous “NA” entries, which limits transparency and reproducibility. The authors should attempt to retrieve missing information, for example by contacting study authors, or explicitly state when details were not reported in the primary papers.
2. The mechanistic discussion is relatively superficial. A more integrated synthesis of how specific flavonoids modulate pathways such as BDNF/CREB, Nrf2, or apoptosis would considerably enhance the scientific contribution of the review.
3. The review does not include a risk-of-bias assessment. Pre-clinical systematic reviews are expected to evaluate study quality using tools such as SYRCLE or CAMARADES. A domain-based appraisal for each study, summarized in a figure or table and discussed in relation to the findings, is essential to strengthen confidence in the conclusions.
4. The translational perspective could be expanded. Beyond noting the absence of clinical trials, the authors should propose specific next steps, such as identifying candidate flavonoids, clarifying dosing strategies, or considering formulation approaches that could guide early human studies.

**Minor Comments**

1. Figure 1 contains inconsistencies between the numbers shown in the flow diagram and those reported in the main text; these should be corrected.
2. The main table would be more informative if it included the direction of change (up- or down-regulation) and the specific flavonoid associated with each effect.
3. Some in-text citations (e.g., [25], [30]) do not correspond to the reference list, suggesting formatting or numbering errors that should be revised.
4. The manuscript treats all behavioral assays as equivalent. Adding a supplementary table that classifies each test by memory domain, validation status, and sensitivity to flavonoid effects would provide important context.

Are the rationale for, and objectives of, the Systematic Review clearly stated?

Yes

Is the statistical analysis and its interpretation appropriate?

Yes

If this is a Living Systematic Review, is the ‘living’ method appropriate and is the search schedule clearly defined and justified? (‘Living Systematic Review’ or a variation of this term should be included in the title.)

Not applicable

Are sufficient details of the methods and analysis provided to allow replication by others?

Yes

Are the conclusions drawn adequately supported by the results presented in the review?

Yes

Reviewer Expertise:

Aging and neurodegenerative disease

I confirm that I have read this submission and believe that I have an appropriate level of expertise to confirm that it is of an acceptable scientific standard, however I have significant reservations, as outlined above.

# Reviewer report: Reviewer response for version 1

**DOI:** 10.5256/f1000research.181373.r408299

**Published:** 2025-09-12

**Authors:** Rajwinder Kaur

**Affiliations:** 1 Chitkara University, Rajpura, Punjab, India

This manuscript is a robust and valuable addition to the literature on natural compounds in cognitive health. With minor additional reporting clarifications, it meets the scientific and methodological standards for systematic reviews and provides a thorough synthesis supporting further translation of flavonoids in amnesia research.

 Following areas can be considered for improvement:

- The review would benefit from a more detailed presentation of excluded studies and reasons for exclusion (e.g., a supplementary PRISMA table).
- While heterogeneity is thoroughly discussed, more structured narrative on the impact of model variations on outcome comparability would enhance clarity.
- Clear mention of review registration (e.g., PROSPERO, if applicable) would strengthen method transparency.Following areas can be considered for improvement:
- While heterogeneity is thoroughly discussed, a more structured narrative on the impact of model variations on outcome comparability would enhance clarity.
- A clear mention of review registration (e.g., PROSPERO, if applicable) would strengthen the method transparency.

Are the rationale for, and objectives of, the Systematic Review clearly stated?

Yes

Is the statistical analysis and its interpretation appropriate?

Yes

If this is a Living Systematic Review, is the ‘living’ method appropriate and is the search schedule clearly defined and justified? (‘Living Systematic Review’ or a variation of this term should be included in the title.)

Not applicable

Are sufficient details of the methods and analysis provided to allow replication by others?

Yes

Are the conclusions drawn adequately supported by the results presented in the review?

Yes

Reviewer Expertise:

Anticancer, neurodegenerative diseases, inflammatory diseases

I confirm that I have read this submission and believe that I have an appropriate level of expertise to confirm that it is of an acceptable scientific standard.

# Reviewer report: Reviewer response for version 1

**DOI:** 10.5256/f1000research.181373.r405522

**Published:** 2025-09-05

**Authors:** Zeenath Banu

**Affiliations:** 1 RBVRR Women's College of Pharmacy, Hyderabad, Telangana, India

This systematic review (2010–2024) synthesizes 25 studies on flavonoids in amnesia.

 Flavonoids enhanced cognition, biochemical markers, and gene expression in animal models through antioxidant, anti-inflammatory, and neurotrophic effects. Following PRISMA 2020, the review details species, interventions, and outcomes; however, it describes animal models but not the specific induction methods of amnesia (e.g., scopolamine, Aβ injection, trauma, ischemia) and the plant names should be written in italics(Table 4).Inconsistent use of in-text citations (sometimes just "Ref. 3")

Are the rationale for, and objectives of, the Systematic Review clearly stated?

Yes

Is the statistical analysis and its interpretation appropriate?

Partly

If this is a Living Systematic Review, is the ‘living’ method appropriate and is the search schedule clearly defined and justified? (‘Living Systematic Review’ or a variation of this term should be included in the title.)

Not applicable

Are sufficient details of the methods and analysis provided to allow replication by others?

Yes

Are the conclusions drawn adequately supported by the results presented in the review?

Yes

Reviewer Expertise:

Neuropharmacology, Neuroscience

I confirm that I have read this submission and believe that I have an appropriate level of expertise to confirm that it is of an acceptable scientific standard.
